# Tailoring Interfacial Bonding in PEEK Composites via Custom Macromolecular Silane Coupling Agents: From Synthesis to Enhanced Thermomechanical Properties

**DOI:** 10.3390/ma19102017

**Published:** 2026-05-12

**Authors:** Jianquan Li, Xiang Li, Ziyong Liang, Huailin Fan, Qingyu Ma

**Affiliations:** School of Materials Science and Engineering, University of Jinan, Jinan 250022, China; mse_lijq@ujn.edu.cn (J.L.);

**Keywords:** poly(ether ether ketone) (PEEK), macromolecular silane coupling agent, composite material

## Abstract

This study addresses the poor compatibility between resin and reinforcement and the weak interfacial bonding in poly(ether ether ketone) (PEEK)-based composites by preparing several macromolecular silane coupling agents. Three types of coupling agents with different structures were synthesized using hydroxyl-terminated PEEK oligomers, and their structures were confirmed by FT-IR, NMR, and XPS analyses. The molecular weights were determined by GPC, and TG analysis showed that all three coupling agents exhibited good thermal stability. Glass fibers and carbon fibers were surface-modified with these coupling agents. SEM and EDS analyses revealed uniform coatings on the fiber surfaces, accompanied by increases in the characteristic elements of the coupling agents. Mechanical tests showed that the tensile and flexural strengths of the treated composites were higher than those of the untreated ones. DSC and TG results indicated significant improvements in crystallinity and thermal properties. These enhancements are attributed to improved fiber–matrix compatibility and interfacial bonding. Overall, this work establishes a structure-tailored macromolecular silane coupling strategy, providing new insights into structure–property relationships and offering an effective approach to enhance the performance of PEEK-based composites.

## 1. Introduction

**Figure 1:**
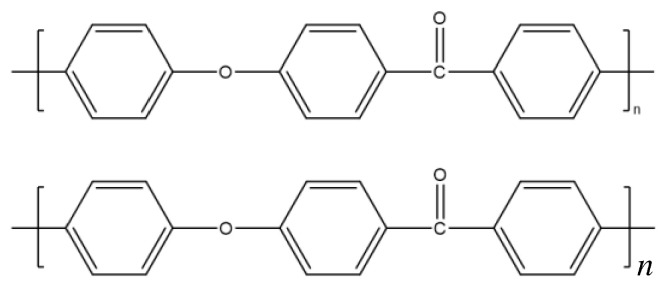
Molecular structure of polyether ether ketone.

Polyether ether ketone (PEEK) is a semi-crystalline aromatic thermoplastic resin and one of the few heat-resistant engineering plastics that can be used above 200 °C. The molecular structure of PEEK resin (Figure 1) determines its unique combination of rigidity and toughness. Composites prepared by fiber blending or filler reinforcement exhibit excellent mechanical properties, high heat resistance, corrosion resistance, solvent resistance, impact resistance, friction resistance, creep resistance, good flame retardancy, and radiation resistance. PEEK is widely applied in high-speed rail systems, new energy vehicles, transportation equipment, aerospace, electronics, information technology, national defense, and other fields, and it is regarded as one of the most promising materials in the 21st century [1,2,3,4,5].

PEEK-based composites [6,7] have been extensively investigated worldwide for many years. However, the inherent structural characteristics of PEEK resin still lead to several technical drawbacks, including poor solubility, high processing temperature, and high melt viscosity. At present, industrial manufacturing technologies for high-performance PEEK-based composites are largely monopolized by overseas enterprises, such as Fokker and TenCate in the Netherlands, Airbus in the European Union, Evonik in Germany, and Boeing and Cytec in the United States. This situation has become a critical bottleneck restricting the independent development of related industries in China. The fundamental issue lies in the insufficient interfacial compatibility and poor bonding efficiency between reinforcing fillers and the PEEK matrix during composite fabrication. Due to the extremely high melt viscosity of PEEK, typically 10^3^–10^4^ Pa·s under conventional processing conditions, it is difficult to achieve uniform wetting and full impregnation of reinforcing fillers within the resin matrix. In addition, the distinct polarity mismatch and molecular chain inertness between PEEK and common reinforcing materials result in poor interfacial compatibility, weak chemical bonding, and low interfacial adhesion strength, which prevent effective reinforcement. Interfacial debonding between fibers and the matrix is widely recognized as one of the dominant failure mechanisms of advanced PEEK structural composites under service loading.

Selection of suitable reinforcement materials and optimization of processing and molding methods are among the approaches used to address the above-mentioned problems. Reinforcement materials include carbon fiber (CF) and glass fiber (GF). In particular, continuous CF-reinforced PEEK composites exhibit the best mechanical properties [8,9,10,11]. Molding processes such as hot stamping, automatic fiber placement, and 3D printing have been developed. Fujihara et al. [12,13] found that the bending strength and interlaminar shear strength (ILSS) of CF/PEEK composites first increased and then decreased with rising molding temperature. This is because the PEEK matrix can degrade with higher processing temperatures and longer holding times, indicating that a suitable molding process requires a lower temperature and shorter holding time. Qureshi et al. [14] investigated the correlation between process parameters and mechanical properties of CF-reinforced PEEK composites fabricated via in situ consolidation. They found that increasing the tape lay-up speed improved the mechanical properties of the composites, whereas increasing the tape spreading speed led to an overall decrease in ILSS. Increasing the temperature enhanced the monolayer shear strength and fracture toughness, but the monolayer shear strength decreased slightly when the composite surface was resin-rich. In addition, because PEEK is a semi-crystalline polymer, heating and cooling during the molding process affect its crystalline state, which in turn influences the material properties. Talbott et al. [15] found that the tensile and compressive properties of unreinforced PEEK are sensitive to processing temperature and that the shear strength, modulus, and fracture toughness of both PEEK and CF-reinforced composites depend mainly on the degree of crystallinity.

From the perspective of interface enhancement mechanisms, improving the compatibility between reinforcing materials and the matrix is the key to solving interfacial problems in composites. Compatibilizers improve interfacial properties, either by pretreating the surface of reinforcing materials before compounding with the matrix or by directly compounding both the reinforcing materials and the matrix simultaneously. This enables chemical bonding at the reinforcement–matrix interface, surface adsorption and wetting, and morphological changes of the polymer in the interfacial region. As a result, the interaction between the reinforcing material and the matrix, that is, the interfacial adhesion, is enhanced, leading to improved mechanical properties of the composites. At present, the most widely used and extensively studied compatibilizers are silane coupling agents, such as aminopropyltriethoxysilane (KH-550) [16] and γ-glycidoxypropyltrimethoxysilane (KH-560) [17]. For example, Pan et al. [18] found that Al_2_O_3_-reinforced PEEK composites treated with coupling agents, such as silanes or titanates, exhibited significantly better flexural and impact strengths than untreated Al_2_O_3_/PEEK composites, with an approximately twofold improvement in performance. However, traditional silane coupling agents are small molecules with short organic chain segments. Owing to the chemical inertness of the PEEK structure, the interaction between silanes and PEEK polymer chains is weak, and their ability to improve the structure and properties of the interfacial layer is limited. Consequently, enhancement of the composite’s mechanical properties remains restricted. In addition, small-molecule silane coupling agents suffer from poor thermal stability.

Preparation of macromolecular silane coupling agents (MSCAs) using small-molecule silanes is an effective approach to address the weak interaction between small-molecule silane coupling agents and the polymer matrix. Owing to their long organic chain segments, MSCAs can diffuse and entangle with the polymer chains of the matrix to form effective interfacial bonds. They can also introduce a flexible interfacial layer between the two phases. This not only strengthens the interfacial bonding but also improves the interfacial deformability under stress, thereby simultaneously enhancing the strength, modulus, and toughness of the composites. The effectiveness of MSCAs has been demonstrated in various polymer-based composites, including polypropylene [19], silicone rubber [20], silicone-containing arylene composites [21], and ceramic/fluoropolymer piezoelectric materials [22]. Liu et al. [19] synthesized a terpolymer of styrene, butyl acrylate, and γ-methacryloyloxypropyltrimethoxysilane (KH-570) as an MSCA via atom transfer radical polymerization to improve the mechanical properties of mica-reinforced polypropylene. They found that MSCAs could diffuse and entangle with the macromolecular chains of polypropylene and that higher molecular weight favored the entanglement effect, enabling the formation of a better interfacial layer. The improvements in material properties were significantly superior to those achieved with small-molecule silanes, and the mechanical properties were remarkably enhanced. Dendritic polysiloxane-based MSCAs used as crosslinking agents effectively improved the mechanical properties of silicone rubber [20]. Park et al. [23] added KH-570-modified polybutadiene to GF/vinyl ester resin and found that the total impact energy of the specimens increased from 8.59 J to 11.60 J after treatment with 0.5 wt% MSCA. Cheng et al. [24] reported that TiN nanoparticles were difficult to disperse uniformly in ethyl acetate. However, after surface modification with a randomly copolymerized and functionalized MSCA, the dispersibility of TiN nanoparticles was significantly improved. When incorporated into a polymer matrix, the well-dispersed nanoparticles formed a stable and effective interfacial layer, thereby enhancing the overall performance and service life of the corresponding thermoplastic elastomers and polymer composites.

However, at present, research on macromolecular silane coupling agents is mainly concentrated in the field of thermoplastics and rubbers, such as polypropylene [25,26,27]. Their application in PEEK composites has not been reported worldwide, and only a few studies have focused on other macromolecular coupling agents or macromolecular sizing agents. Jiang et al. [28] used borate-containing polyarylether ketone to treat the surface of aluminum borate whiskers. The mechanical and crystalline properties of the resulting composites were better than those of the untreated samples. In particular, this treatment improved the linear thermal expansion coefficient of the material at high temperatures and reduced its sliding friction coefficient and volumetric wear rate. These results confirm the effectiveness of macromolecular coupling agents in enhancing the interfacial properties of PEEK composites.

Zhu et al. [29] sized polyimide and loosely stacked carbon nanotubes onto CF surfaces, which enhanced the interaction between CF and PEEK through the synergistic effect of the two components and thus improved the material properties. The interlaminar flexural strength, flexural modulus, and shear strength (ILSS) increased by 63%, 70%, and 71%, respectively, with the ILSS rising from 49 MPa to 80 MPa. Wang et al. [30] proposed using crystalline PEEK itself as a sizing agent, and crystalline PEEK was successfully deposited onto CF surfaces via solution sizing. This method ensured the heat resistance and chemical stability of the composites and significantly improved the interfacial shear strength of CF/PEEK. After optimizing parameters such as the melt index of raw PEEK, molecular weight, and sizing agent concentration, the interfacial shear strength of the reinforced PEEK composites reached 83.13 MPa, representing a 91.5% improvement compared with unsized CF/PEEK. Yan et al. [31] developed a soluble aminated PEEK that was compatible with PEEK matrices and could act as a stress-transfer bridge at the CF/PEEK interface to strengthen interfacial adhesion. Although these effective interfacial enhancement methods did not form chemical bonds at the interfaces like those provided by conventional coupling agents, the PEEK-based sizing or wetting agents introduced on CF surfaces essentially played a “coupling-like role”. Benefiting from the identical structure to the PEEK matrix polymer, the sized CF could readily diffuse and entangle with the matrix or form mechanical interlocking, thereby establishing effective interfacial bonding and improving material properties. These results confirm the effectiveness of high-molecular-weight “coupling” in enhancing the interfacial properties of PEEK composites.

Although PEEK and its analogous polymers exhibit excellent thermal stability, chemical resistance, and mechanical properties, the inherently weak interfacial adhesion between fibers and the PEEK matrix significantly limits the overall mechanical performance of the composites. Therefore, interfacial modification and enhancement of interfacial properties have been chosen as the core research focus in this work.

This paper proposes the design and preparation of a PEEK-based macromolecular silane coupling agent (MSCA). Glass fibers (GFs) and carbon fibers (CFs) were selected as reinforcements. Benefiting from its molecular structure similar to the PEEK matrix, the as-prepared MSCA can readily dissolve and diffuse across the fiber–matrix interface and entangle with the molecular chains of the resin. It can also react with functional groups on the fiber surface to form chemical bonds or hydrogen bonds, thereby effectively enhancing the interfacial properties between the matrix and the reinforcements. By adjusting the structure and molecular weight of the MSCA, the interfacial structure between GF/CF and the PEEK matrix can be regulated and optimized. The relationship between the composition and structure of the interfacial layer and the composite properties was investigated to clarify the corresponding structure–property relationship and ultimately obtain high-performance PEEK-based composites.

## 2. Materials and Methods

### 2.1. Materials

The raw materials and chemical reagents used in this study were sourced as follows: 4,4′-difluorobenzophenone (DFBP) and methylhydroquinone (MeHQ) were purchased from Shanghai Bide Pharmaceutical Technology Co., Ltd.(Shanghai, China) Resorcinol (R), 3-isocyanatopropyltrimethoxysilane (KH901), and dibutyltin dilaurate (DBTDL) were obtained from Macklin Biochemical Technology Co., Ltd (Shanghai China). Furthermore, 3,3′,5,5′-tetramethyl-4,4′-dihydroxybiphenyl (TMDHB), potassium carbonate, and toluene were supplied by Sinopharm Chemical Reagent Co., Ltd (Beijing, China). Except for DFBP, which was purified by distillation prior to use, all other reagents were of analytical grade and used directly as received without further purification. The raw materials used in this study are listed in Table S2.

### 2.2. Synthesis of Macromolecular Silane Coupling Agent

#### 2.2.1. Preparation of Hydroxyl-Capped PEEK Oligomers

**Figure 2:**
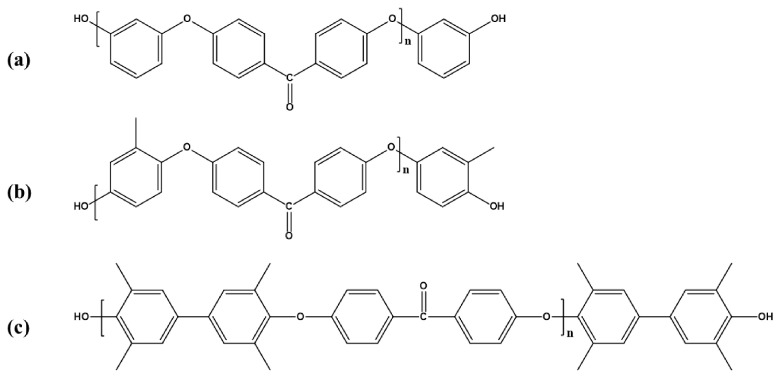
Structures of hydroxyl-capped poly(ether ether ketone) oligomers: structure of R-DFBP (**a**), structure of MeHQ-DFBP (**b**), and structure of TMDHB-DFBP (**c**).

To a four-necked flask, 12.11 g (0.11 mol) of resorcinol and 16.56 g (0.11 mol) of anhydrous potassium carbonate were added, followed by the addition of 30 mL of NMP as the solvent and 25 mL of toluene as the azeotropic agent. The mixture was heated to reflux at 140 °C for 4 h to remove water via azeotropic distillation. After complete dehydration and the removal of toluene, the system was cooled to 100 °C. Subsequently, 21.82 g (0.10 mol) of DFBP was introduced into the flask. The reaction temperature was then elevated to 170 °C and maintained for 6 h. Upon completion, the mixture was cooled to room temperature and precipitated in anhydrous methanol. The resulting solid was collected via centrifugation and filtration and finally dried in a vacuum oven at 120 °C for 24 h. The MeHQ-DFBP and TMDHB-DFBP oligomers were synthesized following an identical procedure. The chemical structure of the resulting hydroxyl-capped PEEK oligomer is illustrated in Figure 2.

#### 2.2.2. Preparation of Macromolecular Silane Coupling Agents

**Figure 3:**
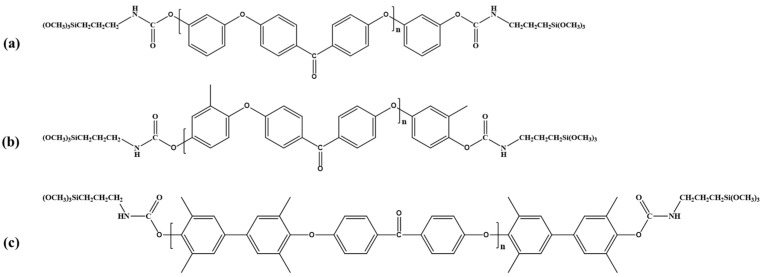
Structures of macromolecular silane coupling agents: structure of R-DFBP-KH901 (**a**), structure of MeHQ-DFBP-KH901 (**b**), and structure of TMDHB-DFBP-KH901 (**c**).

To a four-necked flask, 0.52 g (0.49 mmol) of the PEEK oligomer was added and dissolved in 30 mL of anhydrous tetrahydrofuran (THF). A catalytic amount of dibutyltin dilaurate (DBTDL, 0.5 wt% relative to the reactants) was then introduced. Under a nitrogen atmosphere, 0.34 g (1.46 mmol) of 3-isocyanatopropyltrimethoxysilane (KH901) was added dropwise via a dropping funnel to achieve a functional group molar ratio of 1:1.5. Upon completion of the addition, the reaction mixture was stirred continuously at 70 °C for 4 h. The resulting product was precipitated in anhydrous ethanol, collected by centrifugation, and dried. Depending on the specific oligomer used, the three synthesized macromolecular silane coupling agents were designated as R-DFBP-KH901, MeHQ-DFBP-KH901, and TMDHB-DFBP-KH901. The chemical structure of the coupling agent is illustrated in Figure 3.

### 2.3. Surface Treatment of Fibers with Macromolecular Silane Coupling Agents

Different types of fibers were added separately to a mixed solution of silane coupling agent and NMP. The volume ratio of NMP, relative to the total solution, was 1%, and the mass fraction of silane coupling agent was 2 wt%. A small amount of water was added slowly, and the mixture was heated to 80 °C with continuous stirring and reacted for 12 h. The fibers were collected by filtration, washed with cold NMP, and dried in a vacuum oven at 120 °C for 48 h. All three types of macromolecular silane coupling agents were used to treat four different types of fibers with different specifications in the same manner. The classification and naming of the treated fibers are listed in Table 1.

### 2.4. Preparation of Composites

Fiber/PEEK composites were prepared using a twin-screw extruder. The temperature distribution from the hopper to the mold was 360–380–380–380–370–365–360–360 °C. The fiber content in all composites was fixed at 10%. All materials were dried in an oven at 120 °C for 3 h before molding. Molding was carried out at a mold temperature of 360 °C.

## 3. Results and Discussion

### 3.1. Structural Characterization

#### 3.1.1. Fourier Infrared Spectral Characterization (FT-IR)

**Figure 4:**
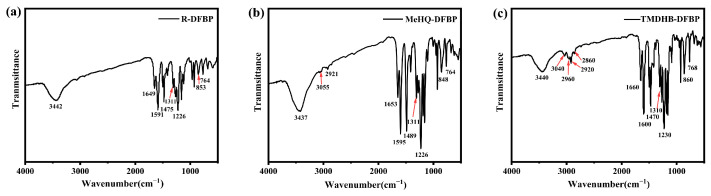
Infrared spectra of hydroxyl-capped polyetheretherketone oligomers: (**a**) R-DFBP, (**b**) MeHQ-DFBP, and (**c**) TMDHB-DFBP.

The infrared spectra of R-DFBP, MeHQ-DFBP, and TMDHB-DFBP (Figure 4a–c) exhibit new carbonyl stretching vibration peaks at 1649, 1653, and 1660 cm^−1^, respectively, which originate from the successful polymerization. The O–H stretching vibration peaks are broad and appear at 3442 cm^−1^, 3437 cm^−1^, and 3440 cm^−1^, respectively. The symmetric stretching vibration peaks of ether bonds are located at 1226 cm^−1^, 1226 cm^−1^, and 1230 cm^−1^. Additionally, the symmetric stretching vibration peaks at 1311 cm^−1^, 1311 cm^−1^, and 1310 cm^−1^ correspond to the C–CO bond stretching and bending vibration peaks. These results confirm that three hydroxyl-capped poly(ether ether ketone) oligomers, namely R-DFBP, MeHQ-DFBP, and TMDHB-DFBP, have been successfully prepared.

**Figure 5:**
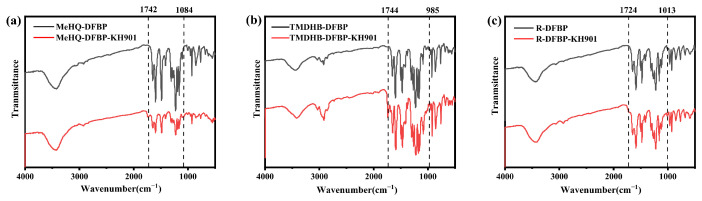
FT-IR spectra of macromolecular silane coupling agents: (**a**) R-DFBP-KH901, (**b**) MeHQ-DFBP-KH901, and (**c**) TMDHB-DFBP-KH901.

The FT-IR spectra of the modified oligomers (R-DFBP-KH901, MeHQ-DFBP-KH901, and TMDHB-DFBP-KH901) and their corresponding hydroxyl-capped PEEK precursors are presented in Figure 5a–c. The appearance of new carbonyl peaks attributed to the formed carbamate groups, along with the newly introduced Si–O absorption bands at 1013, 1084, and 985 cm^−1^, respectively, clearly indicates the successful preparation of the three macromolecular silane coupling agents.

#### 3.1.2. Nuclear Magnetic Resonance Characterization (NMR)

**Figure 6:**
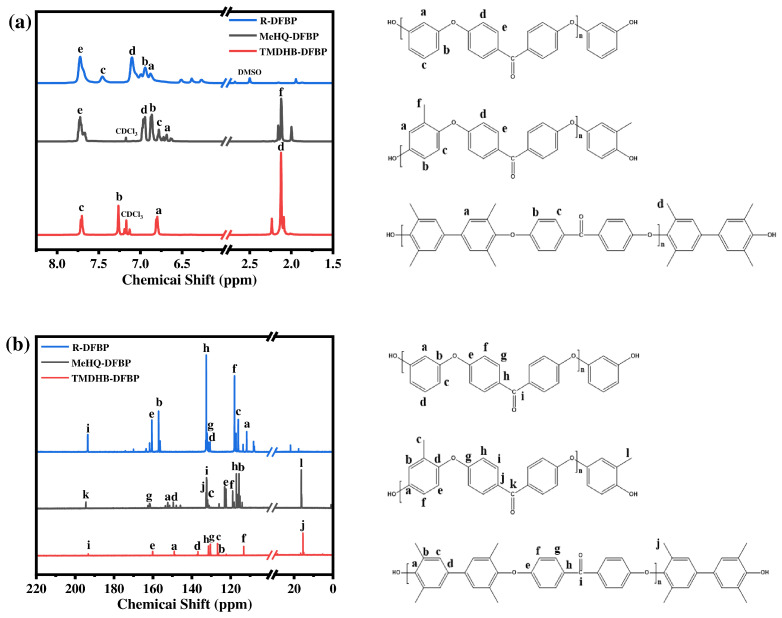
NMR spectra of the three hydroxyl-capped (PEEK) oligomers: (**a**) ^1^H NMR and (**b**) ^13^C NMR.

Figure 6a shows the ^1^H NMR spectra. The characteristic proton peaks of R-DFBP are sequentially labeled a–e. For MeHQ-DFBP, the proton peaks are also assigned as a–e. The methyl protons appear at 2.0–2.3 ppm (labeled f). In TMDHB-DFBP, three characteristic aromatic proton peaks are observed, corresponding to a, b, and c, and the methyl protons similarly appear at 2.0–2.3 ppm (labeled d). Figure 6b displays the ^13^C NMR spectra. The nine characteristic carbon peaks of R-DFBP correspond to the nine distinguishable carbon atoms in the oligomer. The carbonyl carbon signal appears at 194.2 ppm (labeled i), while the other peaks (a–h) are attributed to the aromatic carbon atoms. The spectral analyses for the other two polymers are analogous.

#### 3.1.3. Nuclear Magnetic Resonance Characterization (NMR)

**Figure 7:**
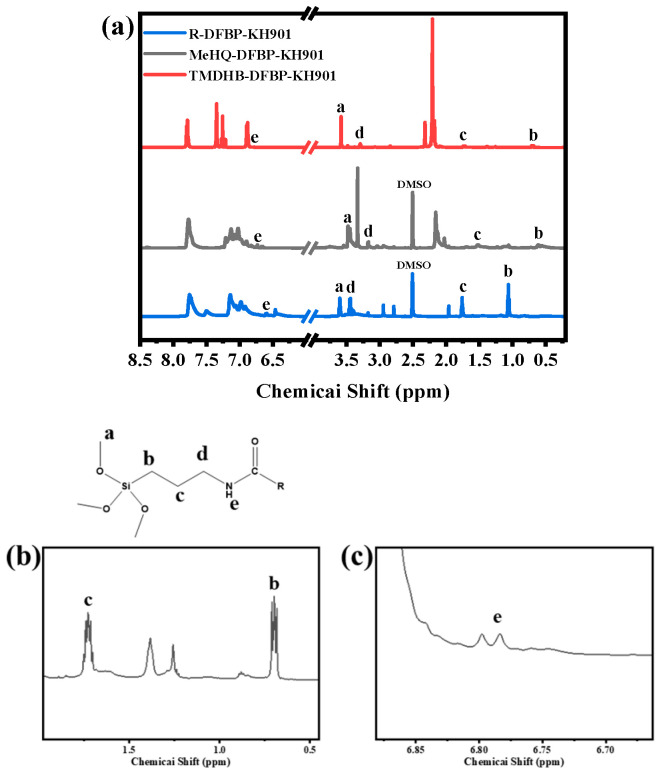
^1^H NMR spectra of the macromolecular silane coupling agents: (**a**) full spectra, and (**b**,**c**) locally magnified regions.

Figure 7 shows the ^1^H NMR spectra of the macromolecular silane coupling agents. As shown in Figure 7a, after coupling with the silane agent, the characteristic peaks of the methyl protons appear at approximately 3.6 ppm (labeled a). The characteristic peaks of three types of methylene protons are observed at around 0.7 ppm (b), 1.7 ppm (c), and 3.8 ppm (d), respectively. The characteristic peak of the amino proton appears at approximately 6.7 ppm (labeled e). Corresponding local magnifications are displayed in Figure 7b. These NMR peak assignments further confirm the successful synthesis and correct structure of the macromolecular silane coupling agents.

#### 3.1.4. XPS Characterization

**Figure 8:**
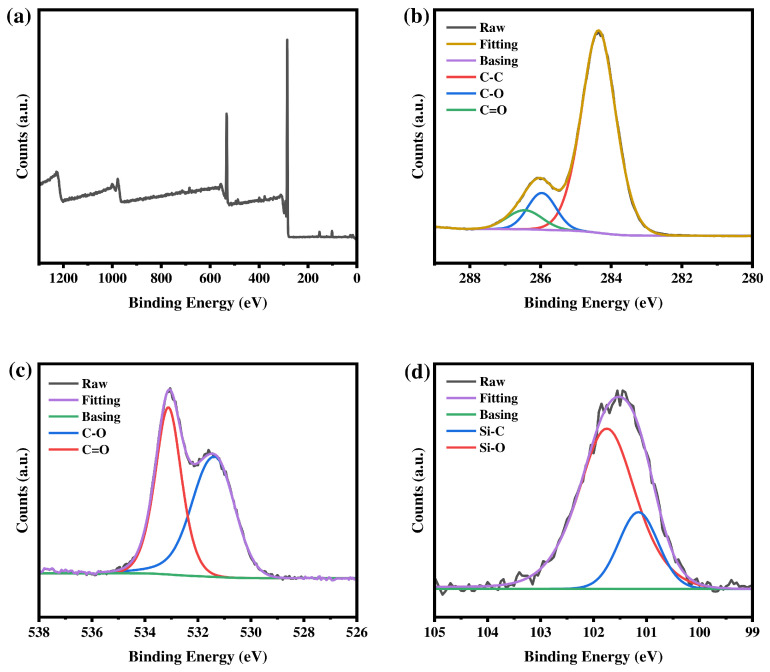
XPS spectra of R-DFBP-KH901: (**a**) survey spectrum and high-resolution spectra of the (**b**) C 1s, (**c**) O 1s, and (**d**) Si 2p regions.

**Figure 9:**
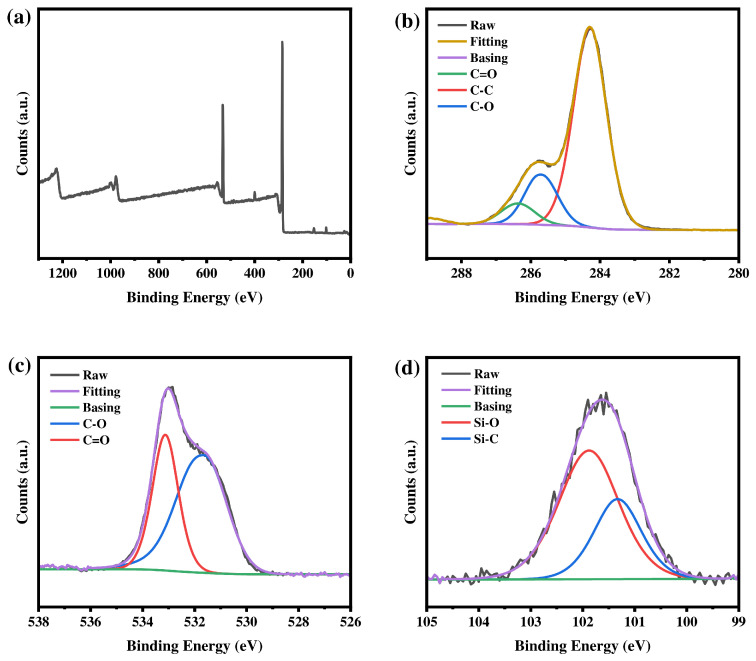
XPS spectra of MeHQ-DFBP-KH901: (**a**) survey spectrum and high-resolution spectra of the (**b**) C 1s, (**c**) O 1s, and (**d**) Si 2p regions.

**Figure 10:**
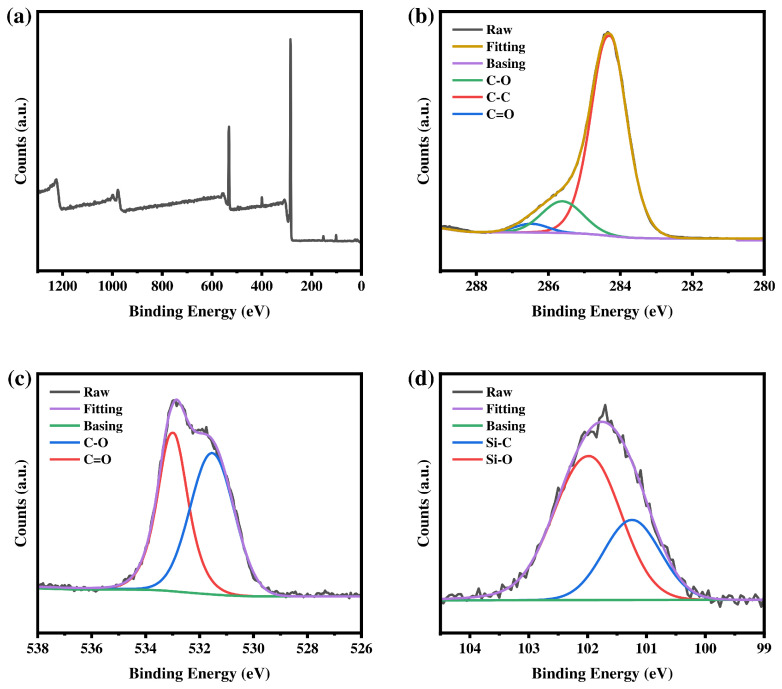
XPS spectra of TMDHB-DFBP-KH901: (**a**) survey spectrum and high-resolution spectra of the (**b**) C 1s, (**c**) O 1s, and (**d**) Si 2p regions.

Figure 8, Figure 9 and Figure 10 show the XPS survey spectra of the three macromolecular silane coupling agents. Four typical characteristic peaks appear at approximately 284 eV (C 1s), 532 eV (O 1s), 399 eV (N 1s), and 101 eV (Si 2p), confirming the presence of the corresponding elements in the macromolecular silane coupling agents. As shown in Figure 8, Figure 9 and Figure 10b–d, high-resolution XPS spectra are fitted to analyze the chemical states of each element. Carbon is mainly present in the forms of C–C (284.7 eV), C–O (286.0 eV), and C=O (286.8 eV). Oxygen is dominated by C–O (532.3 eV) and C=O (533.4 eV). Nitrogen exists mainly as C–N (399.7 eV), and silicon is primarily observed as Si–C (101.5 eV) and Si–O (102.1 eV). The relative atomic percentages of each element in the macromolecular silane coupling agents are listed in Table 2.

### 3.2. Molecular Weight Characterization

In this work, the three synthesized macromolecular silane coupling agents were designed to have 10 repeating units (n = 10). Gel permeation chromatography (GPC, Agilent Technologies, 1260 Infinity II, Santa Clara, CA, USA) was used to characterize their molecular weights. As listed in Table 3, the number-average molecular weights (Mn) of the three PEEK-based macromolecular silane coupling agents—R-DFBP-KH901, MeHQ-DFBP-KH901, and TMDHB-DFBP-KH901—are 3149, 3554, and 4928 g·mol^−1^, respectively.

The test results indicate that the deviations between the experimental number-average molecular weights and the theoretical values are within an acceptable error range. Moreover, the polydispersity index (PDI) of all samples is less than 1.5. Therefore, the actual segment lengths of the as-synthesized macromolecular silane coupling agents are reasonable relative to the targeted theoretical values.

### 3.3. Solubility Characterization

The solubility of the three macromolecular silane coupling agents is summarized in Table 4. All three polymers exhibit excellent solubility in the polar solvent NMP. They are also soluble in DMSO, toluene, and tetrahydrofuran and show slightly slower dissolution in DMF and chloroform. However, they are insoluble in protic solvents such as methanol and ethanol.

### 3.4. Thermal Characterization

**Figure 11:**
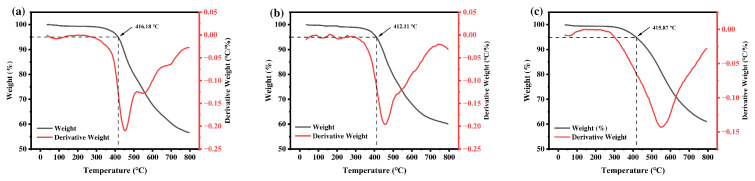
TG and DTG curves of the macromolecular silane coupling agents: (**a**) R-DFBP-KH901, (**b**) MeHQ-DFBP-KH901, and (**c**) TMDHB-DFBP-KH901.

Figure 11a–c show the TG and DTG curves of the three macromolecular silane coupling agents (R-DFBP-KH901, MeHQ-KH901, and TMDHB-DFBP-KH901, respectively). The 5% weight-loss temperatures of the three samples are 416.18 °C, 412.11 °C, and 415.87 °C, respectively. The initial weight loss is attributed to residual solvent from the synthesis process or hydrolysis of methoxy groups during the grafting reaction. All three macromolecular silane coupling agents exhibit favorable thermal stability at the processing temperature of PEEK composites, which generally does not exceed 380 °C.

### 3.5. Surface Morphology Characterization of SCF

**Figure 12:**
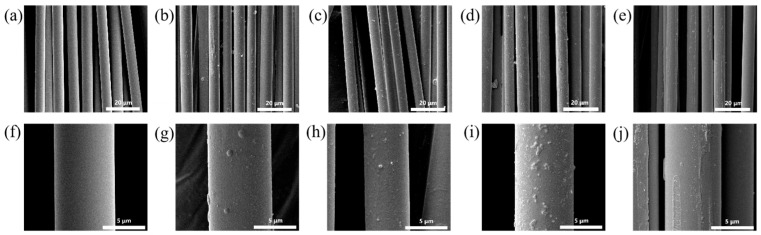
Interfacial morphologies of the modified composites. The images correspond to the following treatments: (**a**,**f**) unmodified control, (**b**,**g**) KH901, (**c**,**h**) R-DFBP-KH901, (**d**,**i**) MeHQ-DFBP-KH901, and (**e**,**j**) TMDHB-DFBP-KH901.

Figure 12 shows representative SEM images of short-cut glass fibers (SGFs) treated with coupling agents. As shown in Figure 12a,f, the surface of glass fibers without coupling agent treatment is smooth and free of any adhesion. Panels (b and g) display the surface morphology of glass fibers treated with the small-molecule silane coupling agent KH901, which appears rough with visible coupling agent adhesion. Panels (c, d, h and i) show the surface of glass fibers coated with a layer of macromolecular silane coupling agent, along with traces of adhesion between fibers. Specifically, the surface of the glass fibers is wrapped with a layer of macromolecular silane coupling agent, and traces of adhesion are observed between adjacent fibers. After treatment, the glass fiber surface becomes rough, with an attached layer and attached particles. These observations indicate that the coupling agent has formed effective binding with the glass fiber surface.

**Figure 13:**
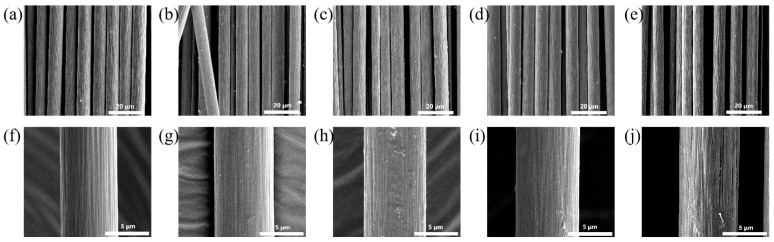
SEM images of the failure cross-sections for composites with different interfacial treatments: (**a**,**f**) coupling agent-free control, (**b**,**g**) KH901, (**c**,**h**) R-DFBP-KH901, (**d**,**i**) MeHQ-DFBP-KH901, and (**e**,**j**) TMDHB-DFBP-KH901.

Figure 13 shows the surface morphology of representative short-cut carbon fibers (SCFs) treated with coupling agents. Panels (a) and (f) display the surface of untreated carbon fibers, which has no surface attachments and exhibits distinct longitudinal grooves. Panels (b) and (g) show the surface morphology of carbon fibers treated with the small-molecule silane coupling agent KH901. The surface grooves become shallower after modification. Panels (c,d) and (h,i) present the SEM images of carbon fibers treated with the three macromolecular silane coupling agents. The original surface grooves are filled by the macromolecular coupling agents, and the fiber surfaces are uniformly coated. As a result, the surface grooves of the carbon fibers almost disappear after treatment.

**Figure 14:**
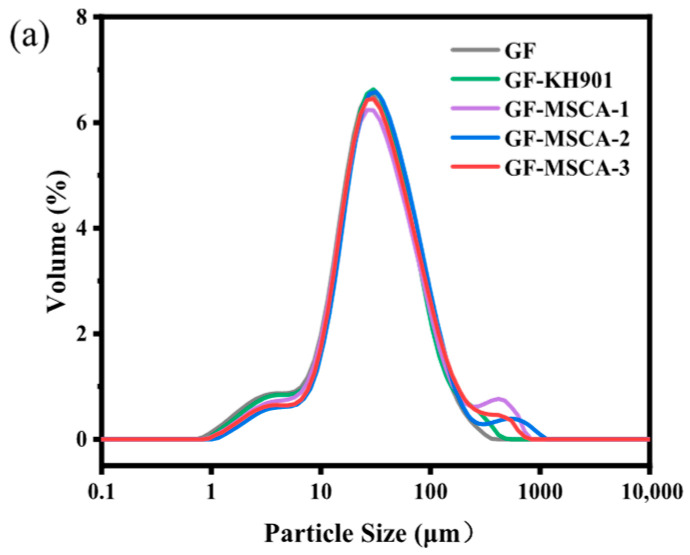

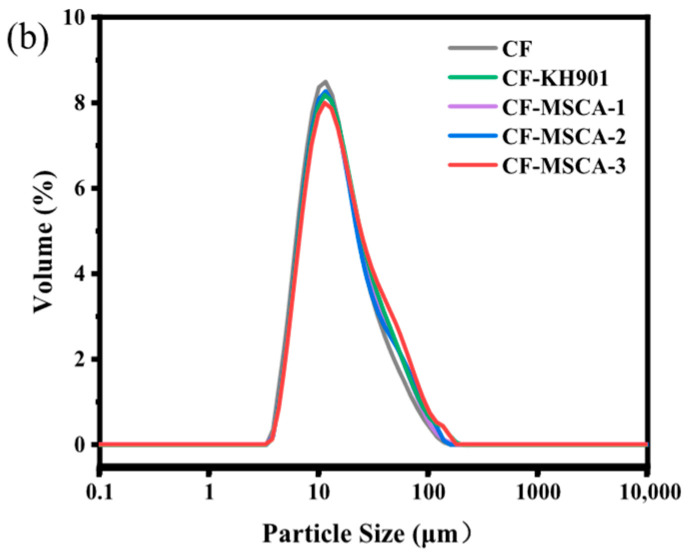
Particle size distributions of the different (**a**) glass and (**b**) carbon fiber powders.

Table 5 and Table 6 show the changes in the average particle size of glass fiber powder and carbon fiber powder treated with different coupling agents, respectively. Combined with the particle size distribution displayed in Figure 14, it can be observed that the average particle size of fibers modified with macromolecular silane coupling agents increases and is higher than that of fibers treated with KH901. After modification with macromolecular silane coupling agents, the average particle size of glass fiber powder increases from 38.4 μm to 57.2, 52.7, and 51.5 μm, respectively, while that of carbon fiber powder increases from 17.8 μm to 19.8, 19.4, and 21.5 μm, respectively. Based on the variation in average particle size, the increase confirms that the fiber surfaces have been successfully coated with the coupling agents, and the coating thicknesses of the three macromolecular silane coupling agents are greater than that of KH901.

Table 7 presents EDS results showing that only O, Si, Mg, Al, and Ca are present on the surface of untreated glass fibers. In contrast, the surface of glass fibers treated with the coupling agent shows increased signals for C and N, which are consistent with the chemical composition of the coupling agent. For untreated carbon fibers, only C and O are detected on the surface, whereas Si and N appear on the surface of carbon fibers after coupling agent treatment. These changes in elemental composition confirm the successful deposition of the coupling agent on the fiber surfaces.

### 3.6. Characterization of Mechanical Properties of Composite Materials

An electrical universal testing machine (ETM305D, Shenzhen Wance, Shenzhen, China) was used for tensile and three-point flexural tests. For the tensile test, dumbbell-shaped specimens were evaluated at a crosshead speed of 2 mm/min. For the three-point flexural test, strip-shaped specimens were tested using a span length of 64 mm and a loading rate of 2 mm/min.

**Figure 15:**
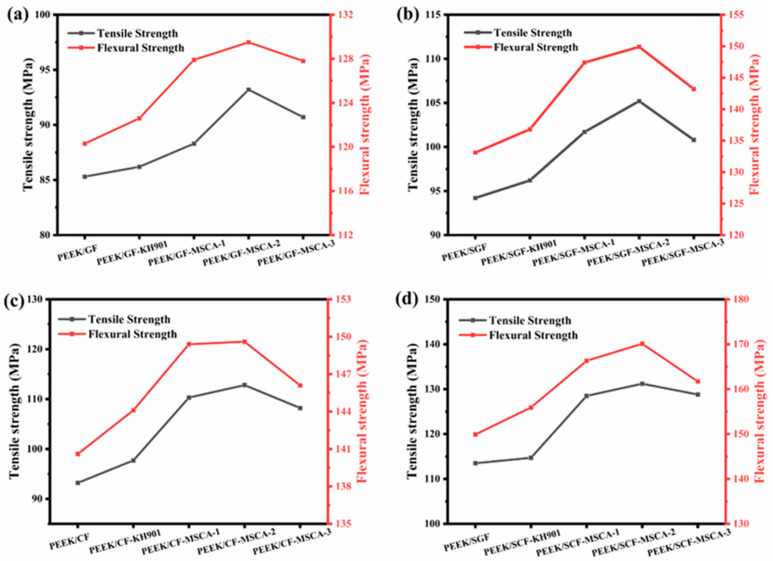
Variations in the mechanical properties of the (**a**) GF/PEEK, (**b**) SGF/PEEK, (**c**) CF/PEEK, and (**d**) SCF/PEEK composites.

The data indicate that the tensile strength, Young’s modulus, flexural strength, and flexural modulus of fiber/PEEK composites treated with small-molecule silane coupling agents are slightly higher than those of untreated composites (Figure 15 and Table 8). Furthermore, the tensile strength, Young’s modulus, flexural strength, and flexural modulus of composites modified with the three macromolecular silane coupling agents are all higher than those treated with small-molecule silane coupling agents.

### 3.7. Characterization of Composite Cross-Section Morphology

**Figure 16:**
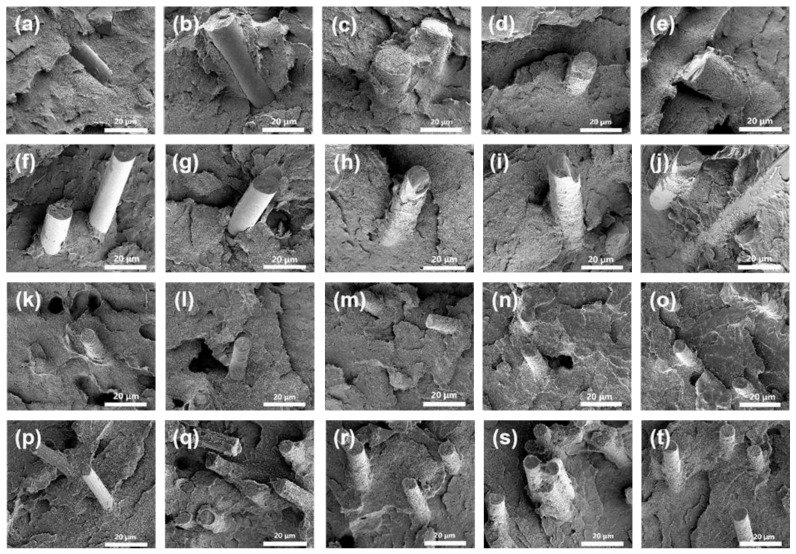
SEM images of the composite failure cross-sections. The images are arranged by composite type: (**a**–**e**) GF/PEEK, (**f**–**j**) SGF/PEEK, (**k**–**o**) CF/PEEK, and (**p**–**t**) SCF/PEEK. For each type, the interfacial treatments from left to right correspond to coupling agent-free control, KH901, R-DFBP-KH901, MeHQ-DFBP-KH901, and TMDHB-DFBP-KH901, respectively.

As shown in Figure 16a,f, the glass fiber surface is smooth with no entanglement of the PEEK matrix. As shown in Figure 16b,g, the glass fiber surface is relatively flat. Owing to the weak interfacial adhesion between the fiber and PEEK resin, partial resin detachment from the fiber surface is observed. For glass fiber/PEEK composites modified with the three macromolecular silane coupling agents, as shown in Figure 16c–e,h–j, both GF (glass fiber powder) and SGF (short-cut glass fiber) surfaces are uniformly embedded in the PEEK matrix. These results demonstrate that the interfacial compatibility between glass fibers treated with the three macromolecular silane coupling agents and the PEEK matrix is significantly improved, leading to excellent interfacial bonding.

As shown in Figure 16k,p, the carbon fiber surface is free of PEEK matrix adhesion, and the inherent groove-like structure of the carbon fiber is clearly observed. As shown in Figure 16l,q, the carbon fiber surface is partially coated with PEEK resin, but the coating uniformity is poor. For carbon fiber/PEEK composites modified with the three macromolecular silane coupling agents, as displayed in Figure 16m–o,r–t, both CF (325-mesh carbon fiber powder) and SCF (3 mm short-cut carbon fiber) are uniformly coated by the PEEK matrix, effectively filling the original surface grooves with no visible fiber exposure. These results confirm that carbon fibers treated with the three macromolecular silane coupling agents also exhibit excellent interfacial bonding with the PEEK matrix.

### 3.8. Characterization of Crystallinity and Thermal Properties of Composites

**Figure 17:**
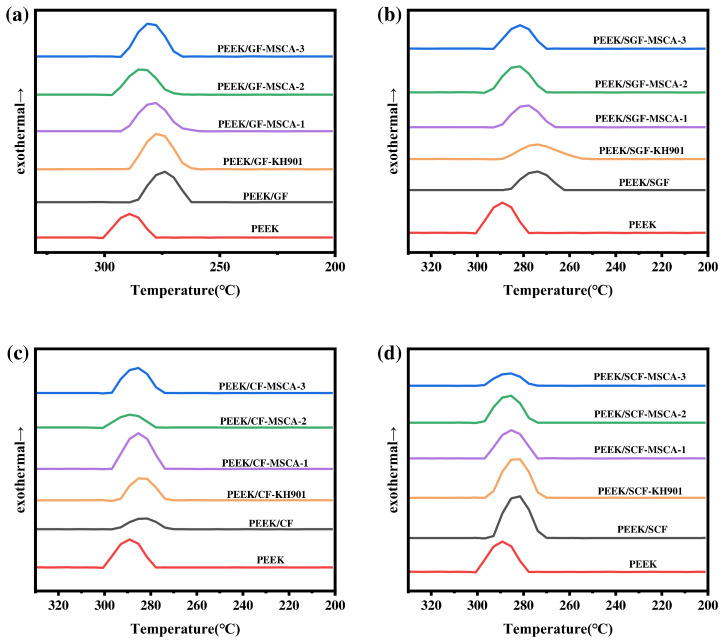
Crystallization peak temperatures (T_c_) of the (**a**) GF/PEEK, (**b**) SGF/PEEK, (**c**) CF/PEEK, and (**d**) SCF/PEEK composites.

The comparative plot of the crystallization peak temperature T_c_ of the composites is shown in Figure 17. The variation in the crystalline properties of the fiber-modified polyether ether ketone composites is summarized in Table 6. The crystallinity $\chi_{c}$ is calculated according to Equation (1).

(1) $$\begin{array}{c} \chi_{c}=\frac{\Delta H_{m}}{∆H_{f}^{0}W_{\mathrm{polymer}}}100\% \end{array}$$

where $\chi_{c}$ represents the degree of crystallinity; $\Delta H_{m}$’ represents the melt temperature of the polyetheretherketone composite, and the values are listed in Table 6; $∆H_{f}^{0}$ represents the melt temperature of the polyetheretherketone at the time of crystalline perfection, with the value of 130 J/g; and $W_{\mathrm{polymer}}$ is the mass fraction of polyetheretherketone in the composite, with the value of 90%.

As can be seen from Table 9, the introduction of fibers contributes to the improvement of the crystallinity of the PEEK, which is attributed to the fact that the introduction of fibers reduces the chain entanglement between the PEEK resin matrices, and the composites with the addition of the macromolecular coupling agent can reach a maximum crystallinity of 31.18%.

The results show that the crystallization peak temperature (T_c_) of the composites shifts to lower values compared with pure PEEK (T_c_ = 291.13 °C). As T_c_ comprehensively reflects the crystallization behavior of the materials, this indicates that the introduction of fibers exerts a certain negative effect on the crystallization of the composites. This effect is mainly attributed to the relatively large particle size of the fibers, which leads to an insignificant heterogeneous nucleation effect when the fibers acted as nucleating agents and hindered the mobility of PEEK chain segments. The T_c_ values of PEEK/GF-KH901, PEEK/SGF-KH901, PEEK/CF-KH901, and PEEK/SCF-KH901 composites slightly increase toward higher temperatures compared with the untreated composites. This increase is explained by the improved interfacial connection between the fiber surface and the PEEK matrix induced by the small-molecule silane coupling agent. After coupling modification, the PEEK molecular chain segments tend to arrange perpendicularly to the interfacial region, which effectively improves the crystallization ability of the PEEK polymer in the interfacial phase.

Among the various fiber/PEEK composites, the crystallization peak temperature (T_c_) values of the three composites modified with macromolecular silane coupling agents are the highest. This suggests that after grafting onto the fiber surface, the macromolecular silane coupling agents contain PEEK-like chain segments and exhibit a molecular structure similar to that of the PEEK matrix, thereby imparting excellent compatibility between the fibers and the PEEK matrix. Such structural similarity and interfacial compatibility not only optimize the interfacial structure and provide effective chain entanglement and interconnection but also exert a lubricating effect that facilitates the molecular mobility of the polymer matrix. Consequently, the T_c_ of fiber-filled PEEK composites treated with macromolecular silane coupling agents is significantly enhanced. These results demonstrate that the introduction of macromolecular silane coupling agents improves the interfacial bonding between fibers and the matrix, thereby effectively enhancing the crystallization properties of the composites.

For the same fiber type, the T_c_ of PEEK/GF (276.09 °C) is higher than that of PEEK/SGF (275.03 °C), and the T_c_ of PEEK/CF (286.31 °C) is higher than that of PEEK/SCF (285.20 °C). This is explained by two competing effects induced by fiber incorporation. One effect is the weakening of intermolecular interactions within the matrix, and the other is the restriction of matrix chain segment motion. Powdered fibers have smaller particle sizes and disperse better in the matrix, thus exerting less steric hindrance on the chain segments and promoting heterogeneous nucleation, which favors crystal formation. In contrast, short-cut fibers have larger dimensions and stronger hindrance effects, leading to a downward shift in T_c_. Among different fiber types, carbon fibers are more conducive to the crystallization of PEEK than glass fibers, resulting in relatively higher T_c_ values for carbon fiber-reinforced composites.

**Figure 18:**
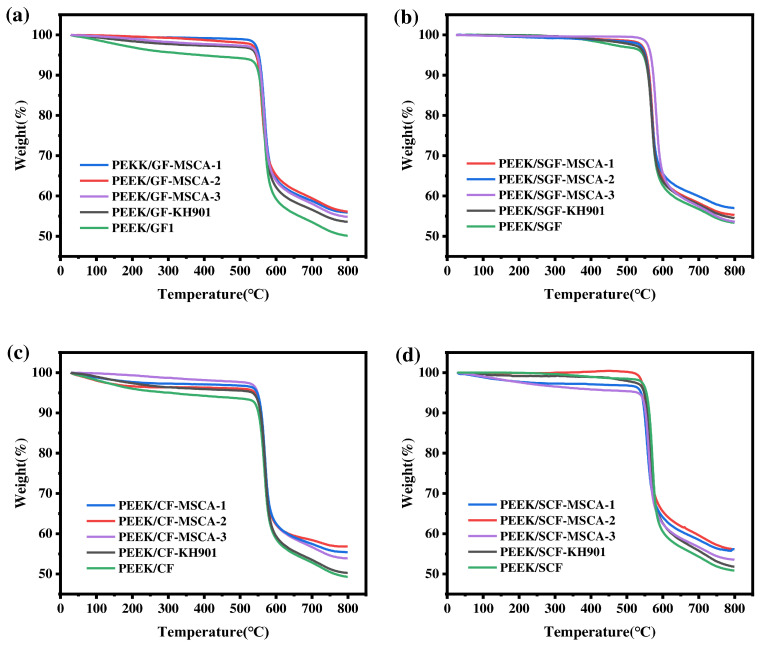
Thermogravimetric TG plot of fiber/PEEK composites (**a**) PEEK/GF, (**b**) PEEK/SGF, (**c**) PEEK/CF, (**d**) PEEK/SGF.

Thermogravimetric analysis curves of the fiber/PEEK composites are presented in Figure 18. The results show that the thermal decomposition of the composites at high temperatures is slightly delayed after fiber modification with macromolecular silane coupling agents. This is attributed to the presence of PEEK-like structural segments in the macromolecular coupling agents, which improves fiber–matrix compatibility and strengthens interfacial bonding between fibers and the PEEK matrix. The enhanced interfacial interaction effectively increases the thermal stability of the composites. All four types of fiber/PEEK composites exhibit excellent thermal stability, with 5% weight-loss temperatures exceeding 500 °C. This indicates that the composites fully satisfy the processing and service temperature requirements of PEEK.

## 4. Conclusions

In this study, three types of structure-tailored macromolecular silane coupling agents were successfully synthesized using hydroxyl-terminated PEEK oligomers. The thermal analysis showed that all three coupling agents exhibited excellent thermal stability, ensuring suitability for high-temperature processing of CF/PEEK composites. Surface modification experiments demonstrated uniform grafting of the coupling agents onto both glass and carbon fibers, as confirmed by SEM and EDS analyses. The treated composites exhibited significant enhancements in mechanical performance, with tensile strength increasing up to 131.2 MPa and flexural strength increasing up to 170.1 MPa, compared with the untreated composites. DSC and TG results indicated that the crystallinity and thermal properties were also notably improved, with crystallinity reaching 31.18%. These improvements are attributed to enhanced interfacial compatibility and bonding between the fibers and the PEEK matrix. Overall, this work provides a novel strategy for designing macromolecular silane coupling agents, offering new insights into interfacial engineering and a practical approach to significantly enhance the performance of PEEK-based composites.

## Figures and Tables

**Table 1:** Naming of fibers treated with coupling agents.

Sample	KH901	R-DFBP-KH901	MeHQ-DFBP-KH901	TMDHB-DFBP-KH901
glass fiber powder	GF-KH901	GF-MSCA-1	GF-MSCA-2	GF-MSCA-3
short-cut glass fiber	SGF-KH901	SGF-MSCA-1	SGF-MSCA-2	SGF-MSCA-3
carbon fiber powder	CF-KH901	CF-MSCA-1	CF-MSCA-2	CF-MSCA-3
short-cut carbon fiber	SCF-KH901	SCF-MSCA-1	SCF-MSCA-2	SCF-MSCA-3

**Table 2:** Types and relative contents of elements contained in silane coupling agents.

Sample	Element	Energy	Relative Content
R-DFBP-KH901	C 1s	284.75 eV	77.73%
	O 1s	532.34 eV	17.64%
	N 1s	399.28 eV	2.35%
	Si 1s	101.82 eV	2.28%
MeHQ-DFBP-KH901	C 1s	284.74 eV	77.61%
	O 1s	532.33 eV	17.76%
	N 1s	399.71 eV	2.47%
	Si 1s	101.82 eV	2.16%
TMDHB-DFBP-KH901	C 1s	284.68 eV	79.92%
	O 1s	532.22 eV	14.89%
	N 1s	399.71 eV	2.71%
	Si 1s	101.91 eV	2.48%

**Table 3:** GPC data for three macromolecular silane coupling agents.

Sample	Number-Average Molecular Weight	Weight Average Molecular Weight	Z-Mean Molecular Weight	Polydispersity
R-DFBP-KH901	3149	4024	5290	1.277866
MeHQ-DFBP-KH901	3554	4617	6212	1.299110
TMDHB-DFBP-KH901	4928	6939	9652	1.408076

**Table 4:** Solubility of three macromolecular silane coupling agents. (++: easy to dissolve; +: partial dissolution; —: does not dissolve).

Sample	NMP	DMF	DMSO	CDCl_3_	Toluene	THF	Methanol	Ethanol
R-DFBP-KH901	++	+	++	+	++	++	—	—
MeHQ-DFBP-KH901	++	+	++	+	++	++	—	—
TMDHB-DFBP-KH901	++	+	++	++	++	++	—	—

**Table 5:** Average particle size of different glass fiber powders (μm).

Sample	GF	GF-KH901	GF-MSCA-1	GF-MSCA-2	GF-MSCA-3
D[4,3]	38.47	42.6	57.2	52.7	51.5

**Table 6:** Average particle size of different carbon fiber powders (μm).

Sample	CF	CF-KH901	CF-MSCA-1	CF-MSCA-2	CF-MSCA-3
D[4,3]	17.8	18.9	19.8	19.4	21.5

**Table 7:** Proportions of different elements on the fiber surface in EDS energy spectra.

Sample	Element (%)						
	C	O	Si	N	Mg	Al	Ca
GF	—	66.41	19.29	—	4.66	4.40	5.24
GF-KH901	4.54	66.12	18.46	3.12	2.31	2.32	3.13
GF-MSCA-1	6.55	64.69	17.56	2.45	2.78	3.54	2.43
GF-MSCA-2	5.87	65.75	17.98	2.99	1.33	2.54	3.54
GF-MSCA-3	7.68	63.67	16.83	1.78	3.31	3.22	3.51
SGF	—	64.22	21.31	—	3.01	3.55	7.91
SGF-KH901	5.21	64.51	20.12	2.78	1.56	2.81	3.01
SGF-MSCA-1	6.55	64.19	19.31	1.98	2.21	1.30	4.46
SGF-MSCA-2	6.89	63.51	17.77	2.04	1.34	2.25	6.20
SGF-MSCA-3	7.45	61.21	18.11	3.01	2.31	2.32	5.59
CF	97.12	2.88	—	—	—	—	—
CF-KH901	89.29	4.01	3.16	3.54	—	—	—
CF-MSCA-1	93.34	3.15	1.52	1.99	—	—	—
CF-MSCA-2	92.23	3.13	2.43	2.21	—	—	—
CF-MSCA-3	94.27	3.09	1.42	1.22	—	—	—
SCF	95.33	4.67	—	—	—	—	—
SCF-KH901	85.11	6.35	4.21	4.33	—	—	—
SCF-MSCA-1	89.78	4.71	3.30	2.21	—	—	—
SCF-MSCA-2	88.21	6.10	3.37	2.32	—	—	—
SCF-MSCA-3	91.01	4.20	2.38	2.41	—	—	—

**Table 8:** Mechanical properties of SCF/PEEK composites treated with different coupling agents.

Samples	Tensile Strength (MPa)	Young’s Modulus (GPa)	Flexural Strength (MPa)	Flexural Modulus (GPa)
PEEK/GF	85.3	2.0	120.3	4.1
PEEK/GF-KH901	86.2	1.9	122.6	4.1
PEEK/GF-MSCA-1	88.3	2.0	127.9	4.3
PEEK/GF-MSCA-2	93.2	2.2	129.5	4.5
PEEK/GF-MSCA-3	90.7	2.1	127.8	4.5
PEEK/SGF	94.2	2.2	133.1	4.4
PEEK/SGF-KH901	96.2	2.2	136.8	4.4
PEEK/SGF-MSCA-1	101.7	2.5	147.4	5.0
PEEK/SGF-MSCA-2	105.2	2.6	149.9	5.1
PEEK/SGF-MSCA-3	100.8	2.5	143.2	4.8
PEEK/CF	93.2	2.1	140.6	4.2
PEEK/CF-KH901	97.7	2.3	144.1	4.6
PEEK/CF-MSCA-1	110.3	2.5	149.4	5.0
PEEK/CF-MSCA-2	112.8	2.7	149.6	5.4
PEEK/CF-MSCA-3	108.2	2.8	146.1	5.4
PEEK/SGF	113.5	2.6	149.9	5.3
PEEK/SCF-KH901	114.7	2.6	155.9	5.4
PEEK/SCF-MSCA-1	128.5	3.6	166.3	7.2
PEEK/SCF-MSCA-2	131.2	3.7	170.1	7.2
PEEK/SCF-MSCA-3	128.8	3.6	161.7	7.0

**Table 9:** Differential scanning calorimeter (DSC) data sheet.

Sample	*T_c_*	*T_m_*	$\Delta H_{m}$	$\chi_{c}$
PEEK	291.13	347.16	31.10	26.58
PEEK/GF	276.09	345.64	31.31	26.76
PEEK/GF-KH901	278.12	345.91	32.10	27.43
PEEK/GF-MSCA-1	281.14	345.09	32.77	28.01
PEEK/GF-MSCA-2	285.08	348.25	34.34	29.35
PEEK/GF-MSCA-3	280.51	346.02	36.33	31.05
PEEK/SGF	275.03	347.03	30.69	26.23
PEEK/SGF-KH901	275.82	345.45	30.74	26.27
PEEK/SGF-MSCA-1	283.68	345.45	31.54	26.96
PEEK/SGF-MSCA-2	284.17	348.85	35.40	30.26
PEEK/SGF-MSCA-3	280.25	344.42	33.72	28.82
PEEK/CF	286.31	345.00	31.16	26.63
PEEK/CF-KH901	285.47	345.75	32.84	28.07
PEEK/CF-MSCA-1	288.07	346.96	34.48	29.47
PEEK/CF-MSCA-2	290.58	345.86	36.48	31.18
PEEK/CF-MSCA-3	286.79	344.58	32.94	28.15
PEEK/SGF	285.20	347.29	30.55	26.11
PEEK/SCF-KH901	285.41	346.29	33.17	28.35
PEEK/SCF-MSCA-1	288.54	343.75	33.52	28.65
PEEK/SCF-MSCA-2	288.80	347.27	35.01	29.92
PEEK/SCF-MSCA-3	286.11	345.96	34.11	29.15

## Data Availability

The original contributions presented in this study are included in the article/Supplementary Material. Further inquiries can be directed to the corresponding author.

## Supplementary Materials

The following supporting information can be downloaded at https://www.mdpi.com/article/10.3390/ma19102017/s1, Figure S1: The average particle size of different powders. (a) glass fiber powders. (b) carbon fiber powders; Figure S2: Photograph of the mechanical testing device for the press machine; Table S1: The mentioned full term and abbreviation; Table S2: Summary of the raw materials and their specifications.
