# Supplementary material for: Tailoring Interfacial Bonding in PEEK Composites via Custom Macromolecular Silane Coupling Agents: From Synthesis to Enhanced Thermomechanical Properties
Source: Materials (Basel). 2026 May 12;19(10):2017. doi: 10.3390/ma19102017 (PMC13208451; doi:10.3390/ma19102017)
Supplement: Supplementary file 1 [file materials-19-02017-s001.zip › materials-4230114-supplementary.pdf]

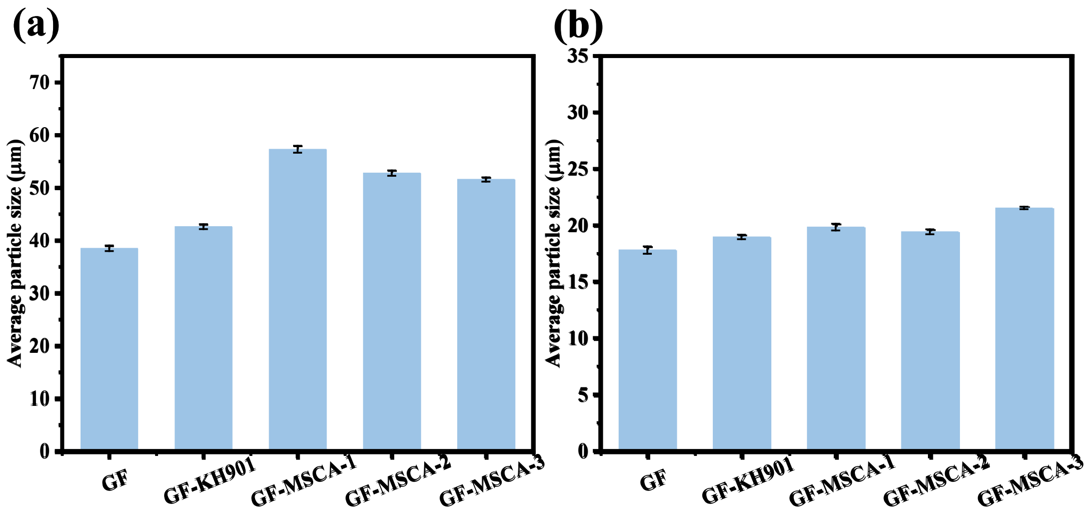

Figure S1. The average particle size of different powders: (a) glass fiber powders; (b) carbon fiber powders.

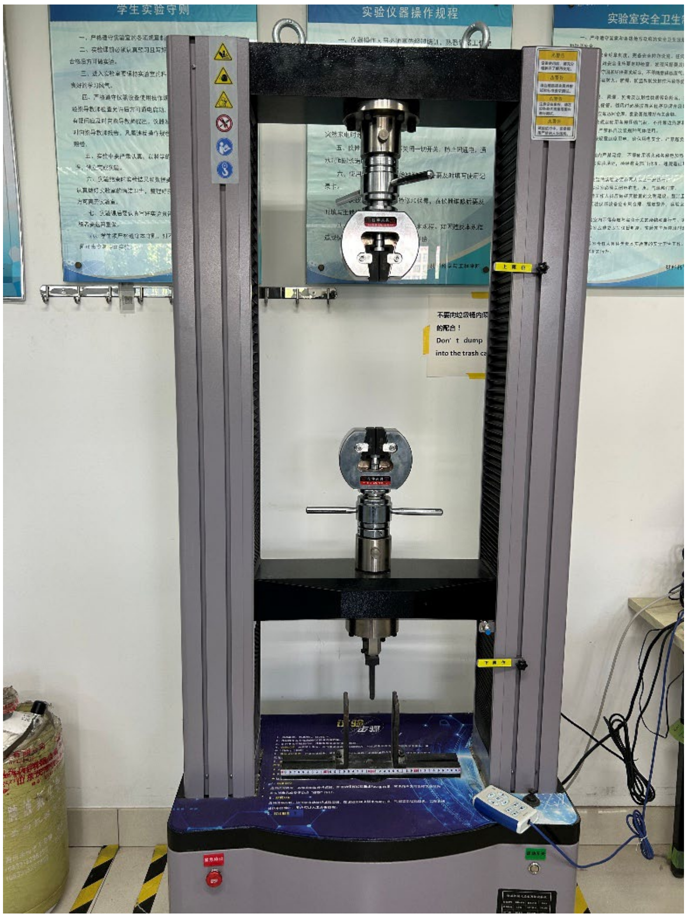

Figure S2. Photograph of the mechanical testing device for the press machine.

Table S1. Full terms and abbreviations.

Full title	Abbreviation
polyether-ether-ketone	PEEK
carbon fiber	CF
interlaminar shear strength	ILSS
aminopropyltriethoxysilane	(KH-550)
γ-glycidoxypropyltrimethoxysilane	(KH-560)
3-isocyanatopropyltrimethoxysilane	(KH901)
polydispersity index	(PDI)
macromolecular silane coupling agents	(MSCA)

Table S2. Summary of the raw materials and their specifications.

Reagent	Abbreviation	Source	Purification
4,4'-Difluorobenzophenone	DFBP	Shanghai Bide Phar-maceutical Technology Co., Ltd.	Purified by distillation
Methylhydroquinone	MeHQ	Shanghai Bide Phar-maceutical Technology Co., Ltd.	Analytical grade
Resorcinol	R	Macklin Biochemical Technology Co., Ltd.	Analytical grade
3-Isocyanatopropyltrimethoxysilane	KH901	Macklin Biochemical Technology Co., Ltd.	Analytical grade
Dibutyltin dilaurate	DBTDL	Macklin Biochemical Technology Co., Ltd.	Analytical grade
3,3',5,5'-Tetramethyl-4,4'-dihydroxybiphenyl	DBTDL	Sinopharm Chemical Reagent Co., Ltd.	Analytical grade
Potassium carbonate	—	Sinopharm Chemical Reagent Co., Ltd.	Analytical grade
Toluene	—	Sinopharm Chemical Reagent Co., Ltd.	Analytical grade
Toluene	—	Sinopharm Chemical Reagent Co., Ltd.	Analytical grade
